# Imbalance between Omega-6 and Omega-3 Polyunsaturated Fatty Acids in Early Pregnancy Is Predictive of Postpartum Depression in a Belgian Cohort

**DOI:** 10.3390/nu11040876

**Published:** 2019-04-18

**Authors:** Axelle Hoge, Valentine Tabar, Anne-Françoise Donneau, Nadia Dardenne, Sylvie Degée, Marie Timmermans, Michelle Nisolle, Michèle Guillaume, Vincenzo Castronovo

**Affiliations:** 1 Department of Public Health, University of Liège, 4000 Liège, Belgium; valentine.tabar@gmail.com (V.T.); afdonneau@uliege.be (A.-F.D.); ndardenne@uliege.be (N.D.); mguillaume@uliege.be (M.G.); 2 Department of Obstetrics and Gynecology, CHR Citadelle Hospital, University of Liège, 4000 Liège, Belgium; docteurdegee@gmail.com (S.D.); marie.timmermans@chu.ulg.ac.be (M.T.); michelle.nisolle@uliege.be (M.N.); 3 Metastasis Research Laboratory, GIGA-CANCER, University of Liège, 4000 Liège, Belgium; vcastronovo@uliege.be

**Keywords:** polyunsaturated fatty acids, erythrocyte, pregnancy, postpartum depression

## Abstract

While studies revealed that the omega-3 polyunsaturated fatty acids (n-3 PUFA) and their mediators would be able to regulate several biological processes involved into the development of postpartum depression (PPD), evidence from observational studies remains mixed. The aim of the present study was to investigate the association between maternal erythrocyte n-3 PUFA, measured in early pregnancy, and the risk of PPD. A Belgian cohort of 72 healthy women was screened. Erythrocyte fatty acids were analysed using gas chromatography. PPD was assessed using the Bromley Postnatal Depression Scale by phone interview one year after delivery. We observed a significant negative association between docosahexaenoic acid (DHA) levels and the risk of postpartum depression in the adjusted model (*p* = 0.034). Higher n-6/n-3 and arachidonic acid (AA)/eicosapentaenoic acid (EPA) ratios were significantly associated with an increased odds of PPD (*p* = 0.013 and *p* = 0.043, respectively). Women with an omega-3 index <5% had a 5-fold increased risk of depressive episode than did those with an omega-3 index ≥5% (OR 5.22 (95% CI 1.24–21.88)). A low n-3 PUFA status, alone and combined with high n-6 PUFA status, in early pregnancy was associated with a greater risk of PPD. Management of maternal n-3 PUFA deficiency can be a simple, safe and cost-effective strategy for the prevention of this major public health issue.

## 1. Introduction

Maternal mental health problems are increasingly recognized as major public health concerns, due to high prevalence rates worldwide and poor outcomes affecting not only mothers but also new-borns and other family members [1]. Postpartum depression (PPD) is the most common complication related to childbearing; approximatively 10 to 20% of mothers experience a depressive episode following delivery in developed countries [2,3]. Beyond maternal suffering [4,5], PPD affects the mother-baby interaction in a negative way [6,7]. This situation is likely to have short and long-term adverse consequences on several aspects of the child development, including emotional, cognitive and behavioural skills [8,9]. Management for mood disorders is challenging due to its multifactorial aetiology, ranging from biological pathways to psychosocial adjustment. The evidence base is still inadequate to fully support clinical decision-making about pharmacotherapy. The effectiveness of antidepressants has been questioned for a few years, and data concerning the prevention of postpartum depression remains very limited [10,11,12]. Furthermore, the benefit-risk ratio associated with depression treatment remains to be further evaluated in pregnant and lactating women [13]. 

Such a situation has drawn attention to lifestyle factors for the prevention and treatment of mental illnesses. The new field of nutritional psychiatry provides compelling support for nutrition as a modifiable risk factor [14]. Omega-3 polyunsaturated fatty acids (n-3 PUFA) are thought to be particularly relevant to mental health as they are critical for brain development and function throughout all stages of life [15]. Notable interest in the role of n-3 PUFA in maternal mental health was triggered by a cross-national ecological study showing an association between both lower seafood consumption and lower docosahexaenoic acid (DHA) content in mothers’ milk and higher rates of postpartum depression [16]. Some observational studies also reported that low n-3 PUFA intake increased the risk of maternal mood disorders [17,18]. Da Rocha et al. found a 2.5-fold higher prevalence of postpartum depressive episodes in women who had a dietary n-6/n-3 ratio greater than 9 in the first trimester of pregnancy [19]. However, other studies have not been able to detect such a link [20,21].

The current literature suggests that the maternal diet does not satisfy the increased demands for long-chain polyunsaturated fatty acids (LCPUFA) during pregnancy and lactation. Food surveys reported insufficient dietary intakes of n-3 LCPUFA among a large portion of European pregnant women [22]. Longitudinal studies focusing on fatty acids markers observed a significant decline in maternal DHA status throughout pregnancy with a slow normalization of postpartum levels [23,24]. Maternal DHA status seems to normalize over a period of 6 months to 1 year after delivery. Subsequent depletion in maternal DHA stores and a lack of recovery postpartum have been associated with an increased risk of postpartum depression [25,26]. Therefore, an omega-3 index (erythrocyte eicosapentaenoic acid (EPA) plus DHA expressed as weight percentage of total fatty acids) lower than 5% in late pregnancy was suggested as a biological risk factor for PPD [27].

There is considerable biological/mechanistic evidence linking n-3 PUFA to mental health. Omega-3 polyunsaturated fatty acids and their mediators would be able to regulate several biological processes involved in the development of depression, including neurotransmission, neuroinflammation and neuroplasticity [1]. DHA content in brain phospholipids contributes to membrane fluidity, which would affect monoamine neurotransmission as well as membrane-bound proteins and cellular signal transduction. In animal models, chronic n-3 PUFA deficiency altered the storage and release processes of serotonergic and dopaminergic neurotransmitters [28]. The sodium-potassium pump (Na^+^/K^+^-ATPase) is an example of a membrane protein whose activity is specifically related to the DHA composition. A disturbance of its activity has been associated with mood disorders [29,30]. 

Long-chain n-3 PUFA are hypothesized to prevent or decrease neuroinflammatory processes which have been more recently connected with depression [31,32]. Many other possible mechanisms, including modulation of gene transcription via peroxisome proliferator-activated receptors (PPARs) and regulation of the production of neuroprotective factors, would also explain the anti-depressant effects of n-3 PUFA. 

While accumulating biological evidence supports the hypothesis that PUFA status plays a role in maternal mental health, results from well-designed observational prospective studies are contradictory and inconsistent [1,33]. Fatty acids have recently been recognised as the nutritional biomarker most frequently related to pre- and postnatal depression, but the number of such studies is still limited [34]. To our knowledge, only five studies [27,35,36,37,38] have assessed maternal erythrocyte fatty acid composition despite it appearing to be the most appropriate measure of long-term incorporation of fatty acids in tissues [39,40].

A particular consideration in designing a cohort study is the timing of exposure measurement. We previously demonstrated that n-3 LCPUFA deficiency could occur as soon as the early stages of pregnancy [41]. Considering the importance of nutrient status for pregnancy outcomes, we tested the hypothesis that low maternal erythrocyte n-3 PUFA levels at the beginning of pregnancy may be a risk factor for postpartum depression. 

## 2. Materials and Methods

### 2.1. Study Population and Design 

The investigation originated from a prospective observational cohort performed in a group of Belgian pregnant women [41]. The main objective was to investigate the relationship between maternal erythrocyte fatty acids and pregnancy and birth outcomes. Enrolment took place in the Obstetrics and Gynaecology Department of the Citadelle Regional Hospital Center, Liège, Belgium between February and August 2016. The women were invited to participate while waiting for their first antenatal appointment. Inclusion criteria were (1) being between the 7th and 18th week of gestation; (2) being free from any chronic diseases such as hypertension and diabetes; (3) presenting with singleton pregnancy. 

**Figure 1:**
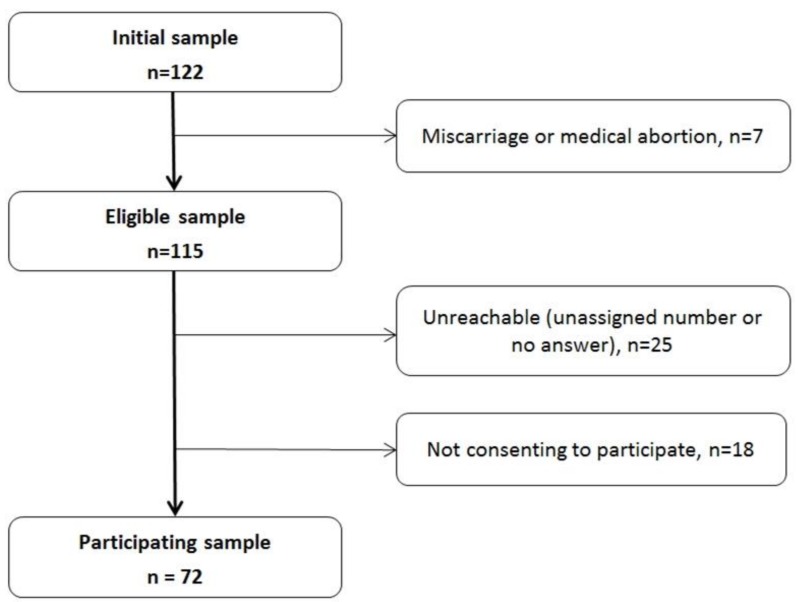
Flowchart of recruitment process.

From the 122 women who enrolled in the initial cohort, 72 (59%) were screened for depressive mood at one year after delivery via phone interviews. Figure 1 represents a flow chart of recruitment process. Of the 122 women, 7 (6%) have been excluded because of miscarriage or medical abortion, 25 (20%) were unreachable and 18 (15%) had refused to take part in the follow-up. 

The study protocol was reviewed and approved by the Ethics Committee of the Citadelle Regional Hospital Center of Liège, Belgium (B412201733757). Verbal informed consent was obtained from all volunteers.

### 2.2. Blood Sampling and Fatty Acid Analysis

Fasting blood specimens were collected on each pregnant woman using an ethylenediaminetetraacetic acid tube, refrigerated and brought in an ice batch to a medical analysis laboratory within 24 h. Samples were centrifuged at 3600 rpm for 5 min to get erythrocytes and plasma separately. The erythrocyte fraction was washed twice with a normal saline solution and was stored at −80 °C until further analysis. The fatty acid composition of erythrocyte membrane phospholipids was measured using a method described previously [41]. Briefly, fatty acids were extracted from erythrocytes and derivatized to fatty acid methyl esters (FAME). FAME were quantified using an Agilent 7890A gas chromatograph (Agilent Technologies Inc., Wilmington, DE, USA).

Analyses generated eight polyunsaturated fatty acid variables: alpha-linolenic acid (ALA), eicosapentaenoic acid (EPA), DHA, total n-3 PUFA, total n-6 PUFA, omega-3 index, n-6/n-3 and arachidonic acid (AA)/EPA ratios. The omega-3 index was defined as erythrocyte EPA plus DHA expressed as weight percentage of total fatty acids [42]. Fatty acids were expressed as percentages of total fatty acids. 

### 2.3. Postpartum Depression

PPD was assessed retrospectively using the Bromley postnatal depression questionnaire [43]. This screening instrument has been specifically designed for detecting previous and current episodes of postnatal depression. 

Mothers were provided with the following description of PPD: “A period of a few weeks or months starting in the first year after the birth of a child when you felt depressed, low spirited or anxious with moments of panic, slept poorly, wept very frequently, daily or almost daily, could not really laugh or enjoy anything, felt irritable and in a poor temper, and felt awful for much of the time”. Researcher then asked if they had experienced such an illness following the last childbirth. Additional questions were related to the duration of the episode (less than one month, 1–3 months, 4–6 months, 7–9 months, 10–12 months, more than 12 months) and the time the depression started and finished. Women were also asked whether they had talked to a health professional, had followed a treatment or had been admitted to a medical unit because of depression during the first year after childbirth. The last section of the questionnaire regarded women’s emotional distress during pregnancy. The English original version of the Bromley questionnaire was translated into French according to standard forward-back translation guidelines [44]. The translated French version was also pre-tested among a group of mothers to ascertain the language accuracy and content clarity. History of depression/maternal depression was self-reported and defined as past diagnosis of depression/maternal depression or current treatment of depression. All phone interviews were conducted by the same trained researcher. Since women from North Africa or The Middle East represented the highest proportion of foreign-nationality women in the initial cohort, an Arabic-speaking interpreter has supported the researcher.

### 2.4. Other Covariates

Sociodemographic, anthropometric and lifestyle characteristics of participants, including age, nationality, education level, socio-professional occupation and relationship status, were collected by a self-administered questionnaire at Baseline. Level of education was briefly expressed as low (primary and lower and upper secondary) and high (non-university degree and university degree). Pre-pregnancy body mass index (BMI) was calculated as weight (kg) divided by height squared (m^2^). Gestational ages at inclusion and at delivery, and parity (at the time of recruitment) were obtained from the medical record. Finally, the pregnant women were invited to communicate any dietary changes made since pregnancy, with a focus on fats. Specifically, questions were “Since pregnancy, have you paid particular attention to your fat intake?—If yes, how?” Detailed information on use of omega-3 polyunsaturated fatty acid supplements was also collected, including product name, type of medication, dose, frequency and duration of use.

Questions regarding psychosocial aspects like planned pregnancy, occurrence of adverse life events during pregnancy and after childbirth, satisfaction regarding social support from entourage during pregnancy and after childbirth, were raised during the telephone interview at one year postpartum. Occurrence of adverse life events was obtained through the question, “Did you suffer any adverse life event during pregnancy/after childbirth”, evaluated according to the women’s perception. Some examples cited included health problems, relationship problems, or any stressful event. Furthermore, smoking status during pregnancy, alcohol consumption during pregnancy, and breastfeeding were included in the interview.

### 2.5. Statistical Methods

Descriptive statistics were used to describe the general characteristics of the women. For quantitative variables, results were expressed as means ± standard deviation (SD). ALA levels were log-transformed to normalise distribution in subsequent statistical analyses. For categorical variables, frequency tables were used. The student’s t-test and Chi-square test were applied to compare mothers who reported postpartum depression and mothers who did not with respect to their characteristics. Same tests were used to compare Baseline characteristics of women who completed the phone interview with those who declined to participate. Binary logistic regression analysis was performed to test the effect of potential risk factors and polyunsaturated fatty acid variables on the incidence of postpartum depression. Models associating PUFA and PPD were adjusted for predictor variables that had statistically significant univariate association with outcome. Results were expressed in terms of the odds ratio (OR) with 95% confidence interval (95% CI). To overcome the problem of quasi-complete separation issue encountered for the satisfaction regarding social support from the entourage, the firth binary logistic regression approach was considered.

The receiver operating characteristic (ROC) curve was generated to identify optimal omega-3 index cut-off in predicting high and low risk of postpartum depression. The area under the curve (AUC) and corresponding 95% CI, sensitivity, specificity and the optimal omega-3 index cut-off using the Youden index (J) were calculated. Statistical analyses were performed using SAS statistical software 9.4 (© SAS Institute Inc., Cary, NC, USA) and R software. Results were considered significant at the 5% critical level (*p* < 0.05). 

## 3. Results

### 3.1. Participants

From the 72 participants, one woman had to interrupt the phone interview and was therefore excluded from analyses. Overall, 17 (23.9%) women had experienced one episode of depression during the first year after the last childbirth. Three reported seeing a health professional, two had followed a treatment and none had been admitted to a medical unit because of depression during the first year after childbirth. Women who did not report any postpartum depression formed the control group (*n* = 54). History of depression/maternal depression was seen in 12 (16.9%) women and was not significantly associated with PPD (*p* = 0.71). 

Women were 29.1 ± 5.00 years old and 47.9% had a low educational level. There were 47 (66.2%) Belgian Caucasians. Table 1 reports characteristics for the depressed and non-depressed participants. We observed a higher frequency of multiparity and socio-professional activity in women who experienced PPD than those who did not (*p* = 0.037 and *p* = 0.045, respectively). The number of participants who reported smoking or alcohol consumption during pregnancy or no breastfeeding was small and not statistically significantly different between groups (Supplementary Table S1). Others studied characteristics were not significantly associated with depressive symptoms. Twenty-nine (40.3%) declared paying particular attention to their consumption of omega-3 fatty acids, by including food naturally rich in or enriched with n-3 PUFA in their diet (*n* = 10) or by using n-3 LCPUFA supplements (*n* = 15) or both (*n* = 4). The proportion of women adopting these favourable dietary practices regarding to n-3 PUFA was not significantly higher in the control group than in those with depression (40.7% vs. 41.2%; *p* = 0.98).

Compared with the women who declined to participate, enrolled women were more likely to be of Belgian nationality (66.2% vs. 22.2%, *p* = 0.0008), to have a high level of education (52.1% vs. 11.1%, *p* = 0.002) and socio-professional occupation (56.3% vs. 16.7%, *p* = 0.003) (Supplementary Table S2).

### 3.2. Determinants of PPD

Of the potential risk factors investigated, there was evidence of an association between adverse life events after childbirth and postnatal onset depression (Table 2). Women who reported adverse life events after childbirth had a four-fold increased risk of depressive mood during the first year than did those without adverse life events (*p* = 0.029). Example of adverse life events after childbirth included mother’s or new-born’s health problems, relationship problems, lack of money or difficult professional circumstances.

### 3.3. Association between PUFA and Odds of Postpartum Depression

Several PUFA variables identified women more likely to experience postpartum depression. Results from unadjusted and adjusted univariate logistic regression analyses are summarized in Table 3. We observed significant associations between DHA, total n-3 PUFA, omega-3 index, n-6/n-3 and AA/EPA ratios and maternal mental health. From a univariate standpoint, higher levels of DHA (*p* = 0.026) and total n-3 PUFA (*p* = 0.027) and higher omega-3 index values (*p* = 0.028) conferred lower odds of postpartum depression. By contrast, higher n-6/n-3 (*p* = 0.006) and AA/EPA (*p* = 0.016) ratios were associated with greater risk of depressive mood. These results were not altered after adjusting for parity, socio-professional occupation and adverse life events after childbirth.

**Figure 2:**
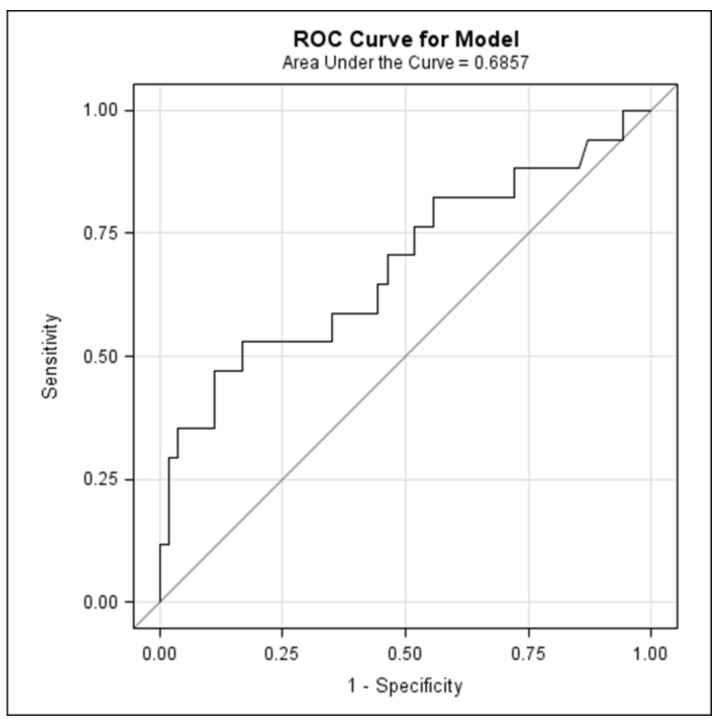
Receiver operating characteristic (ROC) curve of omega-3 index cut-offs as a prediction for postpartum depression.

**Figure 3:**
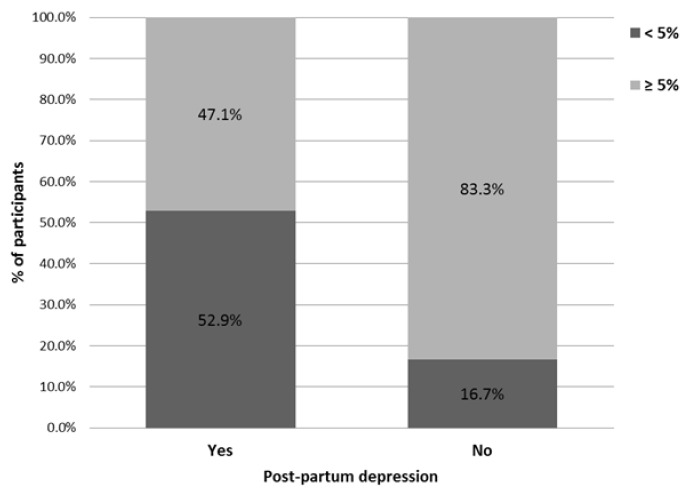
Distribution of participants by omega-index and postpartum depression status.

Figure 2 illustrates the ROC curve of omega-3 index cut-offs as a prediction for postpartum depression. The area under the curve (AUC) was 0.69 (95% CI 0.52–0.85). Optimal omega-3 index cut-off using the Youden index was 5.08%, with a sensitivity of 53% and a specificity of 83.3%. Distribution of women by omega-index and postpartum depression status is displayed in Figure 3. Regression analysis was conducted to examine the OR [95% CI] for the association between optimal omega-3 index cut-off and PPD. From a univariate standpoint, women with an omega-3 index lower than 5% had more than a four-fold increased risk of depressive episode in the first year after childbirth than did those with omega-3 index higher than 5% (OR 4.44 (95% CI 1.35–14.6), *p* = 0.014). Same trend was observed in the adjusted regression model (OR 5.22 (95% CI 1.24–21.88), *p* = 0.024).

## 4. Discussion

The high rate of postpartum depression among the women in this Belgian sample is a worrying result of the current study, with approximately one in four mothers reporting a depressive episode in the first year after childbirth. The Bromley questionnaire is a screening instrument which was specifically designed for detecting PPD retrospectively. Unlike other screening tools commonly developed to identify current episodes a few weeks or months after childbirth, it enables the building up of a longitudinal picture of the illness. Instrument-specific features could explain this result in comparison to the prevalence that is usually reported in developed countries. Another Belgian study using the Edinburgh Postnatal Depression Scale in the first three months after pregnancy has also observed a similar prevalence [45]. Given the adverse short- and long-term effects of PPD on both women and children, these findings highlight an urgent need for further investigation in Belgium.

We found that lower n-3 PUFA content, alone and combined with higher n-6 PUFA content, in maternal erythrocytes at early stages of pregnancy is associated with higher risk of depressive symptoms during the first year after childbirth. Some previous studies have already reported data on fatty acid composition in maternal erythrocytes and their relation to depression postnatally. Such studies showed relationship with health outcomes characterized by less variability and sensitivity to recent intakes than studies using other blood markers [34]. In Norway, Markhus et al. found that DHA content, omega-3 index and n-3/n-6 ratio were all inversely and significantly correlated with the Edinburgh postpartum depression scale score [27]. Similarly, in another study, pregnant women with lower levels of total n-3 PUFA and higher levels of total n-6 PUFA had a higher risk of postpartum depression [38]. By contrast, a large cohort study showed no substantial evidence of association between n-3 LCPUFA and PPD in an adjusted model [35]. 

The largest increase in the risk of PPD was observed for n-6/n-3 ratio. Given the dynamic interactions between omega-3 and omega-6 PUFA, PUFA balance is needed when interpreting evidence for the influence of these fatty acids on health conditions, as recently claimed [46]. PUFA balance is undoubtedly a better indicator of their physiological effects compared to individual PUFA concentrations. More specifically, AA/EPA ratio in cell membranes was related to inflammatory processes occurring during depression [47]. EPA exerts anti-inflammatory actions, partly through antagonizing the activity of pro-inflammatory eicosanoids derived from AA. The close inflammation-depression relationship could be explained in particular by the cytokine-induced activation of the indoleamine 2,3-dioxygenase pathway and, as a consequence, depletion of serotonin in the brain and accumulation of neurotoxic kynurenine metabolites [48]. Some studies have reported a positive correlation between the AA/EPA ratio and the clinical symptoms of depression [49,50] as well as the prevalence of prenatal anxiety [20]. In our study, we found some support for the hypothesis that the balance between AA and EPA is linked with postpartum depression, despite ORs that were relatively small. This association persisted after adjusting model for covariates. Due to the lack of established cut-off values for FA status in pregnant women, we compared our results with laboratory reference AA/EPA values established on healthy adults (previously described in [41]). In our study population, mean AA/EPA values observed in depressed and non-depressed women were much higher than the reference 5.00–10.0 range. Only 3% of the women showed AA/EPA ratio in the laboratory reference values. This functional measure of PUFA status would deserve to be extended in the area of pregnancy health [51]. This topic is particularly relevant in the current context where it is becoming increasingly clear that a pro-inflammatory diet is associated with adverse health outcomes, especially in the field of pregnancy [52]. Associations of high n-6/n-3 and AA/EPA ratios with depression are supported by others studies, as reported above. Overall, these observations are in line with the hypothesis that the changes in Western diet during the last century have contributed to the increase in the incidence of mental disorders through unbalanced PUFA ratios [53]. 

Omega-3 index is gaining interest in mental health [54,55,56,57]. In the present study, we have observed that women with an omega-3 index <5% were approximatively five-fold more concerned by postpartum depression than those with values of 5% or above. Our findings are consistent with one previous research showing a kink in the non-linear inverse omega-3 index—PPD relationship at a level of 5.1% [27]. Such cut-off PUFA values are warranted to identify, as soon as possible, pregnant women who are at increased risk of adverse mental health outcomes as a result of either under or overexposure to fatty acids and those who are not at risk of these outcomes. Together cut-off PUFA values with a standardised measure of PPD could help to obtain consensus on the potential contribution of n-3 PUFA status to maternal mental health.

As far as we know, this is the first study to prospectively investigate the n-3 PUFA status in early pregnancy as a possible risk factor for postpartum depression. It proposes an original contribution focusing on primary prevention. Assuming that LCPUFA levels will continue to decline throughout pregnancy we hypothesised that maternal PUFA status measurement in late pregnancy would produce an even stronger n-3 PUFA—mental health relationship. The measurement of PUFA biomarkers in early pregnancy enables us to observe a sufficient time gap until depression outcome assessment. Since psychological distress could lead to poor dietary quality and lowered PUFA levels, the approach used served to strongly reduce the potential for reverse causality [58]. The current results have clearly highlighted that a little over half of the women who experienced depression could have benefited from the early detection of inadequate PUFA status and consequently, from a prophylactic treatment with n-3 PUFA for optimal mental health outcomes. 

Besides observational studies, supplementation studies also examined n-3 PUFA and their link with maternal depression. However, results are very mixed. As pooled in a recent literature review [59], some trials have demonstrated a positive impact of n-3 PUFA intervention in prevention and treatment of pre- or post-natal depression, but other reported no effect. Currently, no clear conclusions about the effectiveness of n-3 PUFA could be drawn mainly due to methodological heterogeneity between studies. Methodological factors identified are: heterogeneity in study populations (general or medicalized populations) and research protocols (psychiatric diagnosis or self-declaration, composition and dosage of supplements, duration of the trial) as well as differences in assessment of n-3 PUFA exposure (dietary intakes or biomarkers, with or without Baseline status measure, with or without n-6/n-3 ratio) [60]. Fatty acid desaturases (FADS) are key enzymes involved in the conversion of the precursor ALA into long-chain n-3 PUFA derivatives. Recently discovered FADS gene polymorphism could also explain why not all individuals respond in the same way to nutritional interventions [61,62]. In our population, favourable dietary practices regarding n-3 PUFA did not influence the risk of developing PPD. Further analyses showed, however, that participants with such dietary practices had significantly lower n-6 PUFA content and n-6/n-3 and AA/EPA ratios in maternal red blood cells (Supplementary Table S3). These findings suggest that PUFA-PPD relationship was mediated by biochemical measures of PUFA status. By contrast to dietary surveys, nutritional biomarkers provide an objective assessment of dietary exposure. They offer interesting opportunities when conducting research on fatty acids and their health effects, especially since bioavailability of n-3 LCPUFA has been shown to be highly variable [40]. Pending further research in intervention areas, existing data indicate that prenatal supplementation with marine oil not only improves the maternal n-3 LCPUFA status [63] but also that this strategy is safe up to 2 g EPA/DHA per day and well tolerated [64,65]. 

The management of LC n-3 PUFA deficiencies and more widely the promotion of the importance of n-3 LCPUFA consumption early in pregnancy (and ideally in preconception care) could become a key element in preventing PPD. These two major topics should be addressed, at least partially, by front-line caregivers, whose awareness and clinical practices regarding n-3 LCPUFA have previously been demonstrated as rather poor [66]. Clinical guidelines are warranted and could focus primarily on women with habitual low seafood consumption and those at risk of PPD due to other known risk factors. 

The present study has some limitations that should be addressed. The first one is regarding the small sample size observed in our study. However, a *post hoc* power analysis based on erythrocyte n-6/n-3 ratio data, and assuming an alpha error of 0.05, showed a power of greater than 80% (87.6%). Therefore, our sample size appears to have been sufficient to support the conclusions. PUFA status was determined at one time point only. Measuring status also in late pregnancy and measuring changes throughout pregnancy would have made it possible to further consolidate the findings. However, longitudinal status monitoring studies suggest that PUFA concentrations would have been lower at the end of pregnancy [23,24]. Given the multifactorial nature of PPD, several events could have occurred between the exposure and the outcome of interest. To address this limitation, many known risk factors have been studied and included in a multifactorial model, helping to minimize the risk of confounding bias. Cases were defined based on a retrospective self-reported scale rather than on a clinical diagnosis, introducing potential bias in recall. However, the Bromley questionnaire has shown good psychometric properties [43]. Finally, although telephone interviewing has previously been found to be a suitable method for research focusing on depression [67], it did not prevent a higher non-response rate among persons with lower socioeconomic position in the current study. 

## 5. Conclusions

In conclusion, these findings supported the hypothesis that low n-3 PUFA status and imbalance between n-6 and n-3 PUFA status in early pregnancy increase the risk of postpartum depression during the year after childbirth. Prevention of maternal depression should be an important public health priority. Management of maternal n-3 PUFA deficiency can be a simple, safe, and cost-effective strategy at the early stages of pregnancy (and ideally in preconception care). Benefits could be expected not only for the mother, but also the future child, with potential advantages beyond the sphere of maternal mental health alone [68,69]. Faced with a multifactorial disease, n-3 PUFA interventions will probably have to be integrated into a global management, mixing nutritional, psychotherapeutic and/or pharmacological strategies.

## Figures and Tables

**Table 1:** Characteristics of the women (*n* = 71).

Variables	All	Depressive (*n* = 17)	Control (*n* = 54)	*p*-Value ^1^
Age (years)	29.1 ± 5.0	29.5 ± 4.6	28.9 ± 5.1	0.70
Pre-pregnancy body mass index (BMI) class (kg/m²)				0.29
<25	47 (66.2)	9 (52.9)	38 (70.4)	
25–30	20 (28.2)	6 (35.3)	14 (25.9)	
≥30	4 (5.63)	2 (11.8)	2 (3.70)	
Gestational age at inclusion (weeks)	10.6 ± 2.4	10.5 ± 2.6	10.6 ± 2.4	0.97
Gestational age at delivery (weeks)	39.1 ± 1.7	39.2 ± 1.7	39.0 ± 1.8	0.66
Parity				**0.037**
Nulliparous	31 (43.7)	5 (29.4)	26 (48.2)	
Primiparous	26 (36.6)	5 (29.4)	21 (38.9)	
Multiparous	14 (19.7)	7 (41.2)	7 (13.0)	
Nationality				0.31
Belgian	47 (66.2)	13 (76.5)	34 (63.0)	
Other	24 (33.8)	4 (23.5)	20 (37.0)	
Level of education				0.63
Low	34 (47.9)	9 (52.9)	25 (46.3)	
High	37 (52.1)	8 (47.1)	29 (53.7)	
Socio-professional occupation				**0.045**
Yes	40 (56.3)	6 (35.3)	34 (63.0)	
No	31 (43.6)	11 (64.7)	20 (37.0)	
In a relationship				0.21
Yes	60 (84.5)	16 (94.1)	44 (81.5)	
No	11 (15.5)	1 (5.88)	10 (18.5)	

Data are presented as mean ± standard deviation (SD) or number (%). ^1^ *p*-value from Student’s t-test or Chi-square test. Significant *p*-values (<0.05) are in bold.

**Table 2:** Univariate logistic regression model associating postpartum depression (PPD) with potential risk factors. Odds ratio (OR), confidence interval (CI).

Variables	PPD		OR	95% CI	*p*-Value ^1^
	Yes, *n* (%)	No, *n* (%)			
Planned pregnancy					0.71
Yes	13 (81.3)	46 (85.2)	-	-	
No	3 (18.8)	8 (14.8)	1.33	0.31–5.73	
Adverse life event(s) during pregnancy					0.49
No	7 (43.8)	29 (53.7)	-	-	
Yes	9 (56.3)	25 (46.3)	1.49	0.49–4.59	
Adverse life event(s) after childbirth					**0.029**
No	4 (25.0)	31 (57.4)	-	-	
Yes	12 (75.0)	23 (42.6)	4.04	1.15–14.2	
Satisfaction in social support from entourage during pregnancy					0.52 ^2^
Yes	16 (100.0)	50 (92.6)	-	-	
No	0 (0.00)	4 (7.41)	0.34	0.01–9.35	
Satisfaction in social support from entourage after childbirth					0.43 ^2^
Yes	16 (100.0)	49 (90.7)	-	-	
No	0 (0.00)	5 (9.3)	0.27	0.01–6.83	
Emotional distress during pregnancy					0.26
No	6 (37.5)	29 (53.7)	-	-	
Yes	10 (62.5)	25 (46.3)	1.93	0.62–6.07	

Data are presented as number (%). PPD: postpartum depression. ^1^ Significant *p*-values (<0.05) are in bold. ^2^ Results obtained from the firth binary logistic.

**Table 3:** Logistic regression model associating postpartum depression with polyunsaturated fatty acids (PUFA) composition (% of total RBC phospholipid FA).

Fatty Acids (%)	PPD		Unadjusted Model		Adjusted Model ^1^	
	Yes, Mean ± SD	No, Mean ± SD	OR (95% CI)	*p*-Value ^2^	OR (95% CI)	*p*-Value ^2^
ALA	0.14 ± 0.07	0.13 ± 0.04	1.05 (0.25–4.49)	0.95 ^3^	2.31 (0.43–12.5)	0.33 ^3^
EPA	0.46 ± 0.26	0.58 ± 0.26	0.10 (0.00–1.99)	0.13	0.11 (0.00–2.48)	0.17
DHA	4.85 ± 1.36	5.72 ± 1.34	0.55 (0.33–0.93)	**0.026**	0.53 (0.30–0.95)	**0.034**
Total n-3 PUFA	5.45 ± 1.52	6.43 ± 1.52	0.58 (0.35–0.94)	**0.027**	0.57 (0.34–0.98)	**0.043**
Omega-3 index	5.31 ± 1.53	6.30 ± 1.54	0.58 (0.36–0.97)	**0.028**	0.57 (0.34–0.98)	**0.040**
Total n-6 PUFA	24.6 ± 1.63	23.8 ± 1.36	1.51 (1.00–2.26)	0.05	1.55 (0.96–2.51)	0.07
n-6/n-3 ratio	4.92 ± 1.60	3.91 ± 0.94	2.09 (1.24–3.52)	**0.006**	2.31 (1.20–4.45)	**0.013**
AA/EPA ratio	39.3 ± 19.4	28.8 ± 11.3	1.05 (1.00–1.10)	**0.016**	1.05 (1.00–1.11)	**0.043**

PPD: post-partum depression; AA: arachidonic acid; ALA: alpha-linolenic acid; EPA: eicosapentaenoic acid; DHA: docosahexaenoic acid; n-3 PUFA: omega-3 polyunsaturated fatty acids; n-6 PUFA: omega-6 polyunsaturated fatty acids; RBC: red blood cells; FA: fatty acids; AA: arachidonic acid; n-6/n-3 ratio = total n-6 PUFA/total n-3 PUFA; ^1^ Adjusted for parity, socio-professional occupation and adverse life events after childbirth. ^2^ Significant *p*-values (<0.05) are in bold. ^3^ ALA levels were log-transformed to normalise distribution in statistical analyses.

## Supplementary Materials

The following are available online at https://www.mdpi.com/2072-6643/11/4/876/s1, Table S1: Smoking status, alcohol consumption and breastfeeding status of the women (*n* = 71), Table S2: Comparison of the characteristics of the enrolled women (*n* = 71) with the women who declined to participate (*n* = 18), Table S3: Comparison of PUFA composition (% of total RBC phospholipid FA) from the women who reported favourable dietary practices regarding n-3 PUFA intake (*n* = 29) with those who did not (*n* = 42).
