# Supplementary material for: Imbalance between Omega-6 and Omega-3 Polyunsaturated Fatty Acids in Early Pregnancy Is Predictive of Postpartum Depression in a Belgian Cohort
Source: Nutrients. 2019 Apr 18;11(4):876. doi: 10.3390/nu11040876 (PMC6521039; doi:10.3390/nu11040876)
Supplement: Supplementary file 1 [file nutrients-11-00876-s001.zip › Table S1.docx]

**Table S1.** Smoking status, alcohol consumption and breastfeeding status of the women (n=71).

| **Variables** | **All** | **Depressive**  **(n=17)** | **Control**  **(n=54)** | **p-value*** |
| --- | --- | --- | --- | --- |
| **Smoking status during pregnancy** |  |  |  | 0.88 |
| Non-smoker | 62 (88.6) | 14 (87.5) | 48 (88.9) |  |
| Smoker | 8 (11.4) | 2 (12.5) | 6 (11.1) |  |
| **Alcohol consumption during pregnancy** |  |  |  | 0.57 |
| No | 63 (90.0) | 15 (93.8) | 48 (88.9) |  |
| Yes | 7 (10.0) | 1 (6.25) | 6 (11.1) |  |
| **Breastfeeding** |  |  |  | 0.92 |
| Yes | 66 (94.3) | 15 (93.8) | 51 (94.4) |  |
| No | 4 (5.71) | 1 (6.25) | 3 (5.56) |  |

Data are presented as mean ± SD or number (%).

*P-value from Student’s t-test or Chi-square test.
