# Supplementary material for: Imbalance between Omega-6 and Omega-3 Polyunsaturated Fatty Acids in Early Pregnancy Is Predictive of Postpartum Depression in a Belgian Cohort
Source: Nutrients. 2019 Apr 18;11(4):876. doi: 10.3390/nu11040876 (PMC6521039; doi:10.3390/nu11040876)
Supplement: Supplementary file 1 [file nutrients-11-00876-s001.zip › Table S2.docx]

**Table S2.** Comparison of the characteristics of the enrolled women (n=71) with the women who declined to participate (n=18).

| **Variables** | **Participating sample**  **(n=71)** | **Not consenting**  **sample**  **(n=18)** | **p-value*** |
| --- | --- | --- | --- |
| **Age (years)** | 29.1 ± 5.0 | 27.6 ± 6.0 | 0.29 |
| **Pre-pregnancy BMI class (kg/m²)** |  |  | 0.88 |
| < 25 | 47 (66.2) | 13 (72.2) |  |
| 25-30 | 20 (28.2) | 4 (22.2) |  |
| ≥ 30 | 4 (5.6) | 1 (5.6) |  |
| **Gestational age at delivery (weeks)** | 39.1 ± 1.7 | 39.5 ± 1.0 | 0.33 |
| **Parity** |  |  | 0.68 |
| Nulliparous | 31 (43.7) | 8 (44.4) |  |
| Primiparous | 26 (36.6) | 5 (27.8) |  |
| Multiparous | 14 (19.7) | 5 (27.8) |  |
| **Nationality** |  |  | **0.0008** |
| Belgian | 47 (66.2) | 4 (22.2) |  |
| Other | 24 (33.8) | 14 (77.8) |  |
| **Level of education** |  |  | **0.002** |
| Low | 34 (47.9) | 16 (88.9) |  |
| High | 37 (52.1) | 2 (11.1) |  |
| **Socio-professional occupation** |  |  | **0.003** |
| Yes | 40 (56.3) | 3 (16.7) |  |
| No | 31 (43.6) | 15 (83.3) |  |
| **In a relationship** |  |  | 0.90 |
| Yes | 60 (84.5) | 15 (83.3) |  |
| No | 11 (15.5) | 3 (16.7) |  |

Data are presented as mean ± SD or number (%).

*P-value from Student’s t-test or Chi-square test.
