# Supplementary material for: Imbalance between Omega-6 and Omega-3 Polyunsaturated Fatty Acids in Early Pregnancy Is Predictive of Postpartum Depression in a Belgian Cohort
Source: Nutrients. 2019 Apr 18;11(4):876. doi: 10.3390/nu11040876 (PMC6521039; doi:10.3390/nu11040876)
Supplement: Supplementary file 1 [file nutrients-11-00876-s001.zip › Table S3 revised.docx]

**Table S3.** Comparison of PUFA composition (% of total RBC phospholipid FA) from the women who reported favourable dietary practices regarding n-3 PUFA intake (n=29) with those who did not (n=42).

| **Fatty acids (%)** | **Favourable dietary practices** | | **p-value^a^** |
| --- | --- | --- | --- |
|  | **Yes, mean ± SD** | **No, mean ± SD** |  |
| ALA | 0.13 ± 0.03 | 0.13 ± 0.06 | 0.67^b^ |
| EPA | 0.59 ± 0.20 | 0.52 ± 0.30 | 0.30 |
| DHA | 5.81 ± 1.09 | 5.31 ± 1.54 | 0.14 |
| Total n-3 PUFA | 6.52 **±** 1.19 | 5.97 ± 1.76 | 0.12 |
| Omega-3 index | 6.39 ± 1.21 | 5.83 ± 1.78 | 0.12 |
| Total n-6 PUFA | 23.5 ± 1.38 | 24.3 ± 1.44 | **0.02** |
| n-6/n-3 ratio | 3.76 ± 0.95 | 4.42 ± 1.29 | **0.02** |
| AA/EPA ratio | 26.2 ± 8.12 | 34.8 ± 16.5 | **0.005** |

ALA: alpha-linolenic acid; EPA: eicosapentaenoic acid; DHA: docosahexaenoic acid; n-3 PUFA: omega-3 polyunsaturated fatty acids; n-6 PUFA: omega-6 polyunsaturated fatty acids; n-6/n-3 ratio = total n-6 PUFA/total n-3 PUFA

^a^P-value from Student’s t-test

^b^ALA levels were log-transformed to normalise distribution in statistical analyses.
